# Cognitive and brain cytokine profile of non-demented individuals with cerebral amyloid-beta deposition

**DOI:** 10.1186/s12974-021-02169-0

**Published:** 2021-07-04

**Authors:** Lisi Flores-Aguilar, M. Florencia Iulita, Chiara Orciani, Neil Tanna, Jingyun Yang, David A. Bennett, A. Claudio Cuello

**Affiliations:** 1 Department of Anatomy and Cell Biology, McGill University, Montreal, Quebec Canada; 2 Sant Pau Memory Unit, Department of Neurology, Hospital de la Santa Creu i Sant Pau, Biomedical Research Institute Sant Pau, Universitat Autònoma de Barcelona, Barcelona, Spain; 3 Department of Neurology and Neurosurgery, McGill University, Montreal, Canada; 4 Department of Pharmacology and Therapeutics, McGill University, Montreal, Quebec Canada; 5 Rush Alzheimer’s Disease Center, Rush University Medical Center, Chicago, IL USA; 6 Department of Neurological Sciences, Rush University Medical Center, Chicago, IL USA; 7 Department of Pharmacology, Oxford University, Oxford, UK

**Keywords:** Amyloid-beta, Cognition, Cytokines, Alzheimer’s disease, Neuroinflammation, Perceptual speed, No cognitive impairment

## Abstract

**Background:**

Brain inflammation has been increasingly associated with early amyloid accumulation in Alzheimer’s disease models; however, evidence of its occurrence in humans remains scarce. To elucidate whether amyloid deposition is associated with neuroinflammation and cognitive deficits, we studied brain inflammatory cytokine expression and cognitive decline in non-demented elderly individuals with and without cerebral amyloid-beta deposition.

**Methods:**

Global cognition, episodic, working, and semantic memory, perceptual speed, visuospatial ability, and longitudinal decline (5.7 ± 3.6 years) in each cognitive domain were compared between elderly individuals (66–79 years) with and without cerebral amyloid-beta deposition. The expression of 20 inflammatory cytokines was analyzed in frozen temporal, parietal, and frontal cortices and compared between older individuals with and without amyloid-beta deposition in each brain region. Correlation analyses were performed to analyze associations between amyloid-beta load, cytokine expression, and cognitive decline.

**Results:**

Individuals with cortical amyloid-beta deposition displayed deficits and a faster rate of cognitive decline in perceptual speed as compared with those individuals without amyloid-beta. This decline was positively associated with cortical amyloid-beta levels. Elderly individuals with amyloid-beta deposition had higher levels of IL-1β, IL-6, and eotaxin-3 in the temporal cortex accompanied by an increase in MCP-1 and IL-1β in the parietal cortex and a trend towards higher levels of IL-1β and MCP-1 in the frontal cortex as compared with age-matched amyloid-free individuals. Brain IL-1β levels displayed a positive association with cortical amyloid burden in each brain region. Finally, differential cytokine expression in each cortical region was associated with cognitive decline.

**Conclusions:**

Elderly individuals with amyloid-beta neuropathology but no symptomatic manifestation of dementia, exhibit cognitive decline and increased brain cytokine expression. Such observations suggest that increased cytokine expression might be an early event in the Alzheimer’s continuum.

**Supplementary Information:**

The online version contains supplementary material available at 10.1186/s12974-021-02169-0.

## Background

Mounting evidence from human and animal studies indicates that neuroinflammation plays an important role in the development and progression of Alzheimer’s disease (AD). Specifically, epidemiological studies revealed that cognitively healthy individuals under long-term administration of anti-inflammatories have a reduced risk of developing symptomatic AD [1, 2]. The benefit of early anti-inflammatory treatment suggests that a detrimental neuroinflammatory process is active at preclinical AD stages [1, 3, 4]. Indeed, studies in transgenic models of amyloid pathology have demonstrated the presence of a disease-aggravating neuroinflammatory process concomitant with the intraneuronal accumulation of amyloid-beta (Aβ) oligomers [5–9]. While an incipient inflammatory process has been well characterized in AD animal models and has been associated with the abnormal deposition of Aβ, evidence from human studies remains scarce.

In the general population, it has been estimated that about one-third of older non-cognitively impaired individuals (NCI) display brain AD neuropathology [10–13]. Given that aging is the major risk factor for AD and that AD has a long, pre-symptomatic phase where neuropathology starts to ‘silently’ build-up; it is then expected that a proportion of NCI individuals with AD neuropathology should be at the earliest, asymptomatic stages of AD.

Towards elucidating whether incipient AD pathology is associated with inflammatory marker expression and cognitive decline, we analyzed cognitive changes and pro-inflammatory cytokine expression in the temporal, parietal, and frontal cortices of a clinically and neuropathologically characterized cohort of non-demented elderly individuals stratified according to the presence or absence of cerebral Aβ.

Our results indicate that non-demented individuals harboring amyloid pathology display impairments in perceptual speed accompanied by an increase in key cerebral pro-inflammatory markers across different brain regions, strengthening the evidence for an early dysregulation of inflammatory markers at presumable preclinical stages of AD.

## Methods

### Brain tissue

A total of 81 brain tissue samples were obtained from American old clergy members and community-dwelling persons involved in the Religious Orders Study (ROS) and Rush Memory and Aging Project (MAP) [10]. Briefly, ROSMAP comprises community cohorts of aging and AD that enroll cognitively healthy individuals who agree to an annual detailed cognitive evaluation and brain donation at the time of death. For this study, we randomly selected *post-mortem* brain tissue from the temporal (*n* = 28), parietal (*n* = 26), and frontal (*n* = 27) cortices from individuals under age 80 who had been clinically diagnosed as NCI at the last neuropsychological evaluation before death [14]. We excluded cases over age 80 to avoid for potentially confounding effects of ‘SuperAgers’, a group that displays less prominent Alzheimer’s-related changes [15].

### Neuropathological assessment

Neuropathological examination of each case was performed by an experienced pathologist blinded to the clinical diagnosis at the Rush Alzheimer’s Disease Center. A total of 8 brain regions were analyzed per case (hippocampus, entorhinal cortex, midfrontal cortex, inferior temporal, angular gyrus, calcarine cortex, anterior cingulate cortex, and superior frontal cortex). Aβ values for each brain region represent the percentage covered by 4G8 immunoreactivity in 1–2 sections per brain region [10]. The global Aβ value represents the average of 4G8 immunoreactivity across all cortical areas analyzed. Neurofibrillary tangle pathology was determined by the stereological assessment of AT8 immunoreactivity across the entorhinal cortex, CA1, superior frontal cortex, mid frontal cortex, inferior temporal cortex, angular gyrus, cingulate gyrus, and calcarine cortex. Final values represent cortical AT8 density per mm^2^ [10]. Additional neuropathological and genetic information was available, including the modified Consortium to Establish a Registry for Alzheimer’s Disease (CERAD), modified National Institute on Aging (NIA)-Reagan, Braak scores, and APOE genotype [10]. Modified CERAD, NIA-Reagan scores, and Braak scores were based on counts of neuritic plaques and neurofibrillary tangles according to Bielschowsky silver staining. Demographic details are illustrated in Table 1 and in Additional files 1, 2 and 3, Supplemental Tables 1-3.

**Table 1:** Demographics of all the study cases with or without global Aβ deposition

Group		gAβ -	gAβ +	*p-value*
Sample size		*n* = 14	*n* = 16	
Age	range (years)	66.21 – 79.53	67.37 – 79.63	
	mean ± SD	75.83 ± 3.605	75.32 ± 3.708	*0.821*
Sex	(F/M)	6/8	9/7	*0.715*
PMI	range	3 - 29	2.5 – 29.58	
	mean ± SD	11.56 ± 7.238	8.748 ± 7.803	*0.07*
Cogn global	mean ± SD	0.4162 ± 0.3604	0.3103 ± 0.3535	*0.423*
Years of education	mean ± SD	18.29 ± 3.832	17.56 ± 3.245	*0.58*
Apoe4		0	5 (31.25 %)	***0.044***
ApoE distribution	ε 2/2	0	0	
	ε 2/3	2 (14.28 %)	2 (12.5 %)	
	ε 2/4	0	0	
	ε 3/3	12 (85.71%)	9 (56.25 %)	
	ε 3/4	0	5 (31.25 %)	
	ε 4/4	0	0	
Braak score	0 – II	9 (64.29 %)	7 (43.75 %)	*0.298*
	III - IV	5 (35.71 %)	9 (56.25 %)	
Braak score distribution	0	1 (7.14 %)	0	
	I	4 (28.57 %)	4 (25%)	
	II	4 (28.57 %)	3 (18.75 %)	
	III	4 (28.57 %)	5 (31.25 %)	
	IV	1 (7.14 %)	4 (25 %)	
	V	0	0	
	VI	0	0	
CERAD	possible or no AD	14 (100 %)	6 (37.5 %)	***0.0003***
	probable or definite AD	0	10 (62.5 %)	
CERAD distribution	no AD	13 (92.86 %)	5 (31.25 %)	
	possible AD	1 (7.14 %)	1 (6.25 %)	
	probable AD	0	9 (56.25 %)	
	definite AD	0	1 (6.25 %)	
NIA-Reagan	low or no likelihood	14 (100%)	9 (56.25%)	
	intermediate/high likelihood	0	7 (43.75%)	
NIA-Reagan distribution	no likelihood	1 (7.14%)	0	***0.0073***
	low likelihood	13 (92.85%)	9 (56.25%)	
	intermediate likelihood	0	7 (43.75%)	
	high likelihood	0	0	
p-tau	mean ± SD	1.946 ± 2.31	1.274 ± 1.270	*0.918*
Aβ-IR	mean ± SD	0	1.962 ± 1.946	

Abbreviations: *PMI post-mortem* interval, *Cogn* cognition, *gAβ* global amyloid beta, *F* female, *M* male, *IR* immunoreactivity, *SD* standard deviation. Data are presented as mean ± SD

### Immunohistochemical detection and image acquisition of amyloid and tau pathologies in the temporal, parietal, and frontal cortices

Aβ (4G8 immunoreactivity) and tau neuropathology (AT8 immunoreactivity) was assessed as described in [10] at the Rush Alzheimer’s Disease Center. Z-stack Brightfield images of cortical laminae III and V from the temporal, parietal, and frontal cortices were taken with a Zeiss AxioImager M2 Imaging microscope equipped with an AxioCam 506 color digital camera (Zeiss, Canada), and Zeiss ZenPro software v.2.3 (Zeiss, Canada) was used. One representative brain section per region per case was imaged.

### Cognitive assessment

Global cognitive function was calculated using a battery of 19 cognitive tests to assess five domains of cognitive function: (i) episodic memory (word list memory, recall and recognition, east Boston story immediate and delayed, and logical memory Ia immediate and IIa delayed tests), (ii) semantic memory (Boston naming, verbal fluency, and reading tests), (iii) working memory (digit span forward and backward, and digit ordering tests), (iv) perceptual speed (symbol digits modality, number comparison, and Stroop color naming and reading), and (v) visuospatial ability (judgment of line orientation, and standard progressive matrices tests). A composite score of each cognitive domain was obtained by converting raw scores to z scores using the baseline mean and standard deviation of all persons in the ROSMAP studies. The z scores were averaged to yield a final composite score representing global cognitive function [10].

#### Estimated slopes from the random-effects model for each cognitive domain

A linear mixed-effects model with each cognitive domain (episodic memory, semantic memory, working memory, perceptual speed, and visuospatial ability) as the longitudinal outcome was utilized to estimate the person-specific rate of change in each cognitive domain over time (random slope) [16]. The mean time of follow-up for the cognitive evaluation was 5.7 ± 3.6 years (interquartile range: 3–9 years). The statistical model controls for age at baseline, sex, and years of formal education. Cognitive differences in domain-specific cognition, and the rate of cognitive decline were analyzed within the NCI population. As various brain regions are involved in the different cognitive domains that were assessed, we stratified the NCI population according to their global Aβ deposition score. Thus, those with positive values of global Aβ deposition were classified as gAβ+ and those without Aβ deposition were classified as gAβ− (Table 1).

### Segregation of NCI individuals according to Aβ pathology

NCI individuals were segregated into two groups depending on the presence or absence of Aβ in each specific cortical area that was analyzed (temporal, parietal, and frontal cortices). Therefore, those who had zero amyloid immunoreactivity in the examined cortical area (Additional files 1, 2 and 3, Supplemental Tables 1-3) were classified as Aβ−, whereas those harboring Aβ deposition were classified as Aβ+.

### Brain cytokine analysis

Fifty milligrams of frozen brain tissue per case was homogenized in ice-cooled lysis buffer (50 mM Tris, 150 mM NaCl, 0.05% Tween-20, 5 μl per mg of tissue) with 2× phosphatase (PhosStop, Roche) and protease inhibitors (Complete Mini, Roche). Lysed samples were centrifuged at 13,000 rpm for 45 min at 4 °C. To avoid repeated freeze and thaw cycles, supernatants were aliquoted and stored at – 80 °C. Protein concentration was measured using the DC^TM^ Protein Assay Kit (BioRad). Inflammatory protein expression was measured in brain homogenates using an electrochemiluminescence-linked multiplex immunoassay (Meso-Scale Discovery). Three different plates were customized to measure the following inflammatory proteins: Interleukin (IL)-1β, IL-4, IL-6, IL-8, IL-10, IL-13, Tumor necrosis factor (TNF)α, Interferon (IFN)γ, (plate 1), IL-1α, IL-5, IL-7, IL-12p40, IL-15, IL-16, Vascular endothelial growth factor (VEGF)-A, (plate 2) and Eotaxin-3, Interferon-inducible protein (IP)-10, Macrophage inflammatory protein (MIP)-1β, Monocyte chemoattractant protein (MCP)-1, Macrophage-derived chemokine (MDC), Thymus and activation-regulated chemokine (TARC) in plate 3. Samples were normalized to 3 μg/μl in lysis buffer with 1% blocker A (Meso-Scale Discovery) and 1× of protease and phosphatase inhibitors. Samples were loaded in duplicates according to the manufacturer’s recommendations and averaged for analysis. The expression levels of each cytokine were fitted to its corresponding standard curve. Signal was measured with a SECTOR Imager 6000 reader (Meso-Scale Discovery), and data were processed with the Discovery Workbench 4.0 software. VEGF-A signal was below the lower limit of detection, and consequently, there is no data presented in this study.

### Statistical analysis

Data are presented in boxplots where the median is represented by the horizontal line and the whiskers represent the 25th and 75th percentiles. Two-tailed Student’s or Mann–Whitney tests were used for two-group comparisons, according to data normality analysis. Data points above or under 1.5 times the interquartile range were considered as outliers and excluded from analyses. For three-group comparisons, one-way ANOVA or Kruskal–Wallis tests were used followed by Tukey or Dunn’s post hoc tests, respectively. Fisher’s exact test was used to assess differences in sex, APOE genotype, Braak score, CERAD, and NIA-Reagan criteria. Pearson’s or Spearman’s correlation coefficients were assessed in correlation analyses. For correlation and partial correlation analyses, data were log-transformed. Partial correlation analyses of cytokine expression and Aβ or p-tau were controlled for post-mortem interval. Linear regression analyses of cognitive decline and cytokine expression were adjusted for age at death and years of education. No correction for multiple comparisons was performed. Data were analyzed with Graph Pad Prism 8. Partial correlation and linear regression analyses were performed using R version 4.0.4. Significance was set at *p < 0.05.*

## Results

**Fig. 1:**
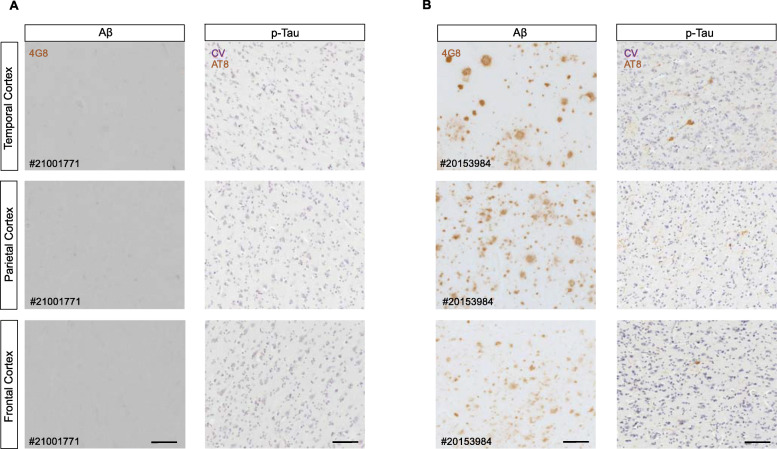
Alzheimer’s disease neuropathology in the temporal, parietal, and frontal cortices of elderly individuals. Representative micrographs depicting amyloid-beta and tau pathology as revealed by immunohistochemistry (IHC) with 4G8 and AT8 antibodies, respectively. **a** Representative micrograph of a case without Aβ deposition in the temporal, parietal, and frontal cortices. **b** Representative micrograph of a case with Aβ deposition in the temporal, parietal, and frontal cortices. *CV* cresyl violet counterstaining. Scale bar = 100 μm

Demographic and neuropathological characteristics of elderly individuals aged 66 to 79 years with or without Aβ deposition are illustrated in Table 1 and Additional files 1, 2 and 3, Supplemental Tables 1-3. Representative images of Aβ and tau histopathology are illustrated in Fig. 1 and in Additional file 4, Supplemental Figure 1. No differences in global cognition, age, sex, years of education, tau pathology, and post-mortem interval were found between groups. The APOE e4 allele was enriched in cognitively normal individuals with global Aβ deposition (*p = 0.044*). Individuals with cerebral Aβ met a neuropathological diagnosis of AD according to the modified NIA-Reagan (*p = 0.007*) and CERAD (*p = 0.0003*) criteria. Cognitive decline and brain inflammatory protein expression were assessed in elderly individuals with no symptomatic manifestation of AD.

### Elderly individuals with Aβ deposition display faster cognitive decline

**Fig. 2:**
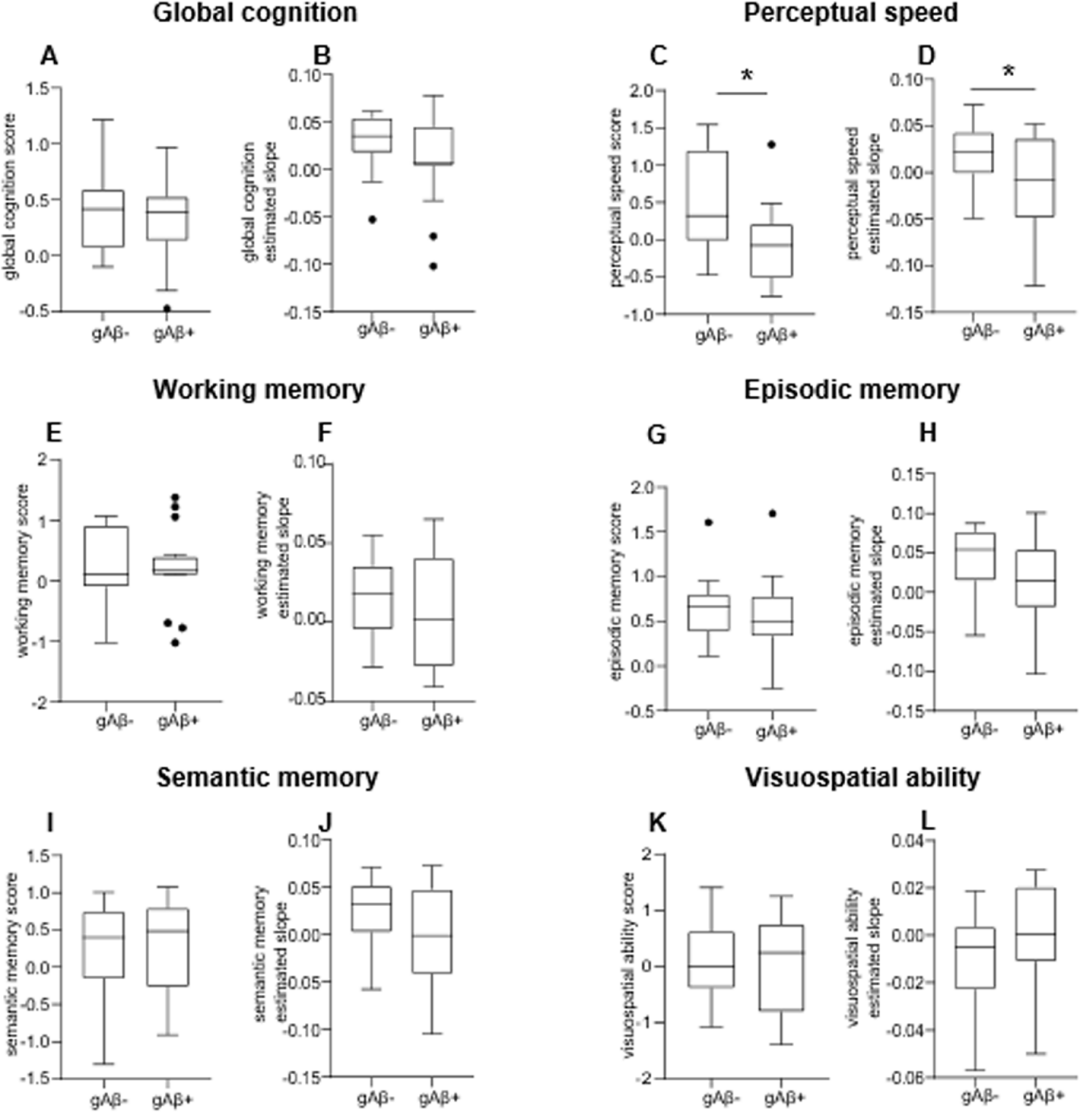
Elderly with Aβ deposition display cognitive deficits. **a**–**l** Cognitive scores and estimated slopes reflecting the person-specific rate of cognitive decline within a mean follow-up period of 5.7 ± 3.6 years (interquartile range: 3–9 years). **c**–**d** Elderly individuals with Aβ deposition display deficits in perceptual speed and a faster rate of cognitive decline in this domain, t (28) = 2.68, *p = 0.012* and t (26) = 2.15, *p = 0.04*, respectivel*y*. **a**–**b** and **e–l** Global cognition, working memory, episodic memory, semantic memory, and visuospatial ability did not differ between elder individuals with or without Aβ deposition. Data are displayed in boxplots where the median is represented by the horizontal line and the whiskers represent the 25th and 75th percentiles; **a**, **c**–**f**, and **h–l** two-tailed Student t test; **b**, **g** two-tailed Mann–Whitney test; Aβ−, n = 14; Aβ+, n = 16; *g* global. Full circles represent outliers. ^*^p < 0.05

Although elderly individuals harboring global Aβ deposition displayed a similar global cognitive score to those without cerebral Aβ (Fig. 2a–b), those with global Aβ deposition displayed deficits in perceptual speed (*p = 0.012)* (Fig. 2c). When assessing the person-specific rate of change (i.e., cognitive decline slope), a higher rate of cognitive decline in perceptual speed (*p = 0.04*) was observed in individuals with Aβ deposition compared with those free of amyloid (Fig. 2d). No other cognitive domains differed between groups (Fig. 2e–l). Notably, a faster decline in perceptual speed was associated with higher Aβ deposition (*p = 0.047*), and no associations were found among cognitive scores and global tau deposition (Additional file 5, Supplemental Table 4).

### Differential upregulation of inflammatory mediators across brain regions in elderly individuals with Aβ pathology and its association with cognitive decline

Inflammatory protein expression was analyzed in the temporal, parietal, and frontal cortices of individuals grouped by the presence or absence of Aβ pathology (Aβ+ and Aβ−, respectively) in each cortical brain area (Additional files 1-3, Supplemental Tables 1-3). Given that some cases displayed long post-mortem intervals, the correlation of cytokine expression in each brain region with the corresponding post-mortem interval was analyzed. IL-4 and TNF-α levels in the temporal cortex and eotaxin-3 and IL-4 expression in the parietal cortex, negatively correlated with the post-mortem interval (Additional file 6, Supplemental Table 5). No associations between cytokine expression and post-mortem interval were found in the frontal cortex (Additional file 6, Supplemental Table 5).

**Fig. 3:**
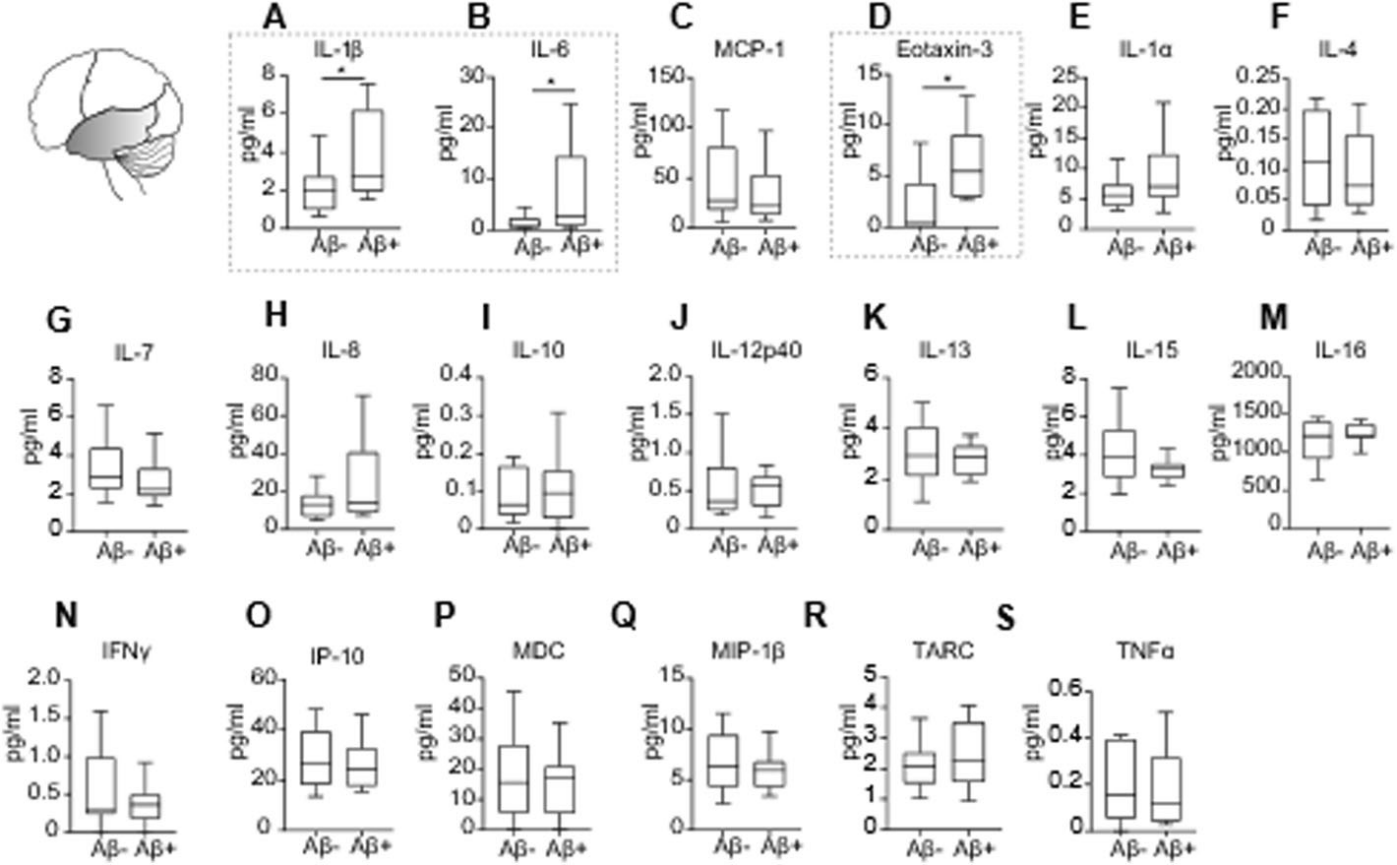
Upregulation of inflammatory cytokines in the temporal cortex of Aβ+ individuals. **a**–**s** Cytokine expression assessed by multispot immunoassays revealed increased levels (dotted line) of **a** IL-1β, two-tailed Student t test; t (24) = 2.403, *p = 0.024*; Aβ−, n = 14; Aβ+, n = 12; **b** IL-6, two-tailed Student t test; t (24) = 2.56, *p = 0.017*; Aβ−, n = 14; Aβ+, n = 12 and D eotaxin-3, two-tailed Student t test; t (22) = 3.594, *p = 0.001*; Aβ−, n = 14; Aβ+, n = 10, in the temporal cortex of non-demented individuals with Aβ deposition as compared with individuals without Aβ deposition in this brain region. **c**, **e**–**s** Expression of other cytokines (MCP-1, IL-1α, IL-4, IL-7, IL-8, IL-10, IL-12p40, IL-13, IL-15, IL-16, IFNγ, IP-10, MDC, MIP-1β, TARC, and TNFα) did not differ between Aβ− and Aβ+ individuals. Data are displayed in boxplots where the median is represented by the horizontal line, and the whiskers represent the 25th and 75th percentiles. ^*^p < 0.05

In the temporal cortex, IL-1β (*p = 0.024*), IL-6 (*p = 0.017*) and eotaxin-3 (*p = 0.001*) were significantly elevated in Aβ+ individuals when compared to those without Aβ deposition (Fig. 3a, b, d). Furthermore, the expression levels of IL-1β and IL-6 positively correlated to the extent of Aβ deposition in this brain region (Additional file 7, Supplemental Table 6). No associations between tau deposition and cytokine expression were found in the temporal cortex (Additional file 7, Supplemental Table 6).

Cognitive decline in episodic, semantic, and working memory was associated with higher IL-1α, IL-13, and MDC levels in the temporal cortex, respectively (Additional file 8, Supplemental Table 7).

**Fig. 4:**
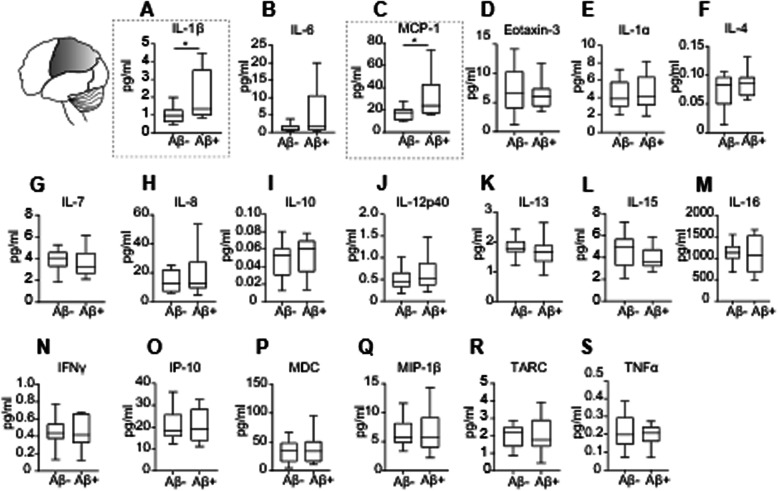
Increased expression of inflammatory cytokines in the parietal cortex of Aβ+ individuals. **a**–**s** Cytokine expression assessed by multispot immunoassays revealed increased levels (dotted line) of **a** IL-1β, two-tailed Student t test; t (22) = 2.46, *p =0.022*; Aβ−, n = 12; Aβ+, n = 12; **c** MCP-1, two-tailed Mann–Whitney test; U = 29, *p = 0.042*; Aβ−, n = 12; Aβ+, n = 10, in the parietal cortex of non-demented individuals with Aβ deposition as compared with individuals without Aβ deposition in this brain region. **b**, **d**–**s** Expression cytokine levels that did not differ between Aβ− and Aβ+ individuals (IL-6, eotaxin-3, IL-1α, IL-4, IL-7, IL-8, IL-10, IL-12p40, IL-13, IL-15, IL-16, IFNγ, IP-10, MDC, MIP-1β, TARC, and TNFα). Data are displayed in boxplots where the median is represented by the horizontal line and the whiskers represent the 25th and 75th percentiles. ^*^p <0.05

Individuals with Aβ positivity in the parietal cortex displayed higher levels of the pro-inflammatory molecules IL-1β (*p = 0.022*) and MCP-1 (*p = 0.042*) (Figure 4a, c). Notably, Aβ deposition positively correlated with IL-1β and MDC levels (Additional file 7, Supplemental Table 6). Tau deposition in the parietal cortex negatively correlated with IL-10 and TNF-α levels and was positively associated with MDC expression levels (Additional file 7, Supplemental Table 6).

In the parietal cortex, higher MIP-1β and TARC levels were associated with a lower rate of cognitive decline in working and episodic memory, respectively (Additional file 8, Supplemental Table 7).

**Fig. 5:**
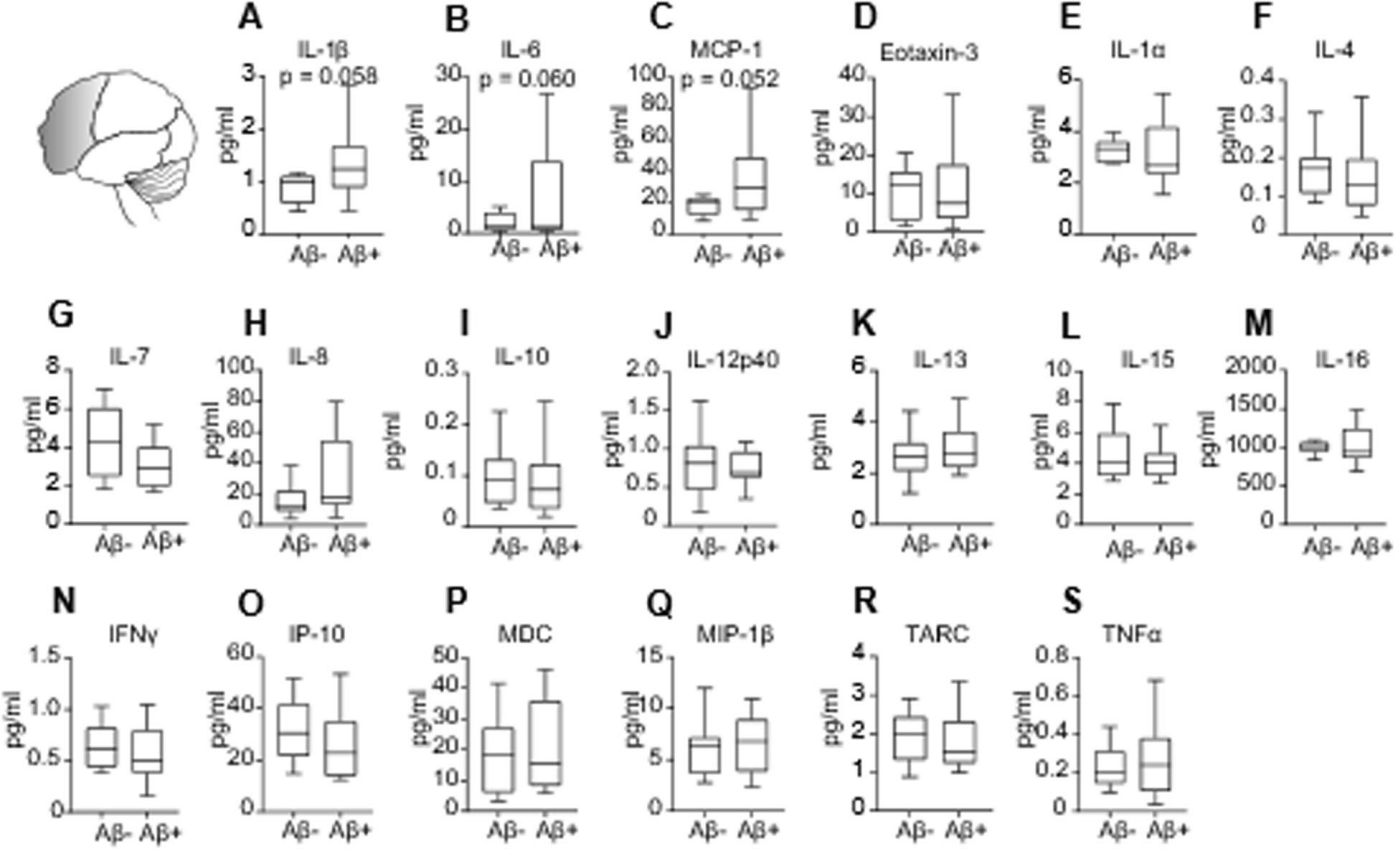
Increased cytokine expression in the frontal cortex of Aβ+ individuals. Cytokine expression assessed by multispot immunoassays revealed a trend towards increased levels of **a** IL-1β, two-tailed Student t test; t (17) = 2.028, *p = 0.058*; Aβ−, n = 9; Aβ+, n = 10; **b** IL-6, two-tailed Student t test; t (22) = 1.981, *p = 0.06*; Aβ−, n = 11; Aβ+, n = 13; and **c** MCP-1, two-tailed Student t test; t (19) = 2.067, *p = 0.052*; Aβ−, n = 9; Aβ+, n = 12, in the frontal cortex of non-demented individuals with Aβ deposition as compared with individuals without Aβ deposition in this brain region. **d**–**s** Expression cytokine levels did not differ between Aβ− and Aβ+ individuals (Eotaxin-3, IL-1α, IL-4, IL-7, IL-8, IL-10, IL-12p40, IL-13, IL-15, IL-16, IFNγ, IP-10, MDC, MIP-1β, TARC, and TNFα). Data are displayed in boxplots where the median is represented by the horizontal line and the whiskers represent the 25th and 75th percentiles

Changes in inflammatory protein expression followed a similar pattern in the frontal cortex, revealing trends toward higher levels of IL-1β, IL-6, and MCP-1 in non-demented individuals with Aβ deposition (*p = 0.058*, *p = 0.060*, *p =0.052*, respectively) (Fig. 5 A–C). Moreover, IL-1β, IL-16, and IL-13 expression positively correlated with Aβ deposition (Additional file 7, Supplemental Table 6). Cytokine expression levels did not correlate with tau deposition in the frontal cortex (Additional file 7, Supplemental Table 6).

Finally, better cognitive scores in visuospatial orientation and perceptual speed were associated with higher IL-16 and IP-10 levels in the frontal cortex (Additional file 8, Supplemental Table 7).

### Neuropathological differences among cortical regions of elderly individuals with and without Aβ deposition

**Fig. 6:**
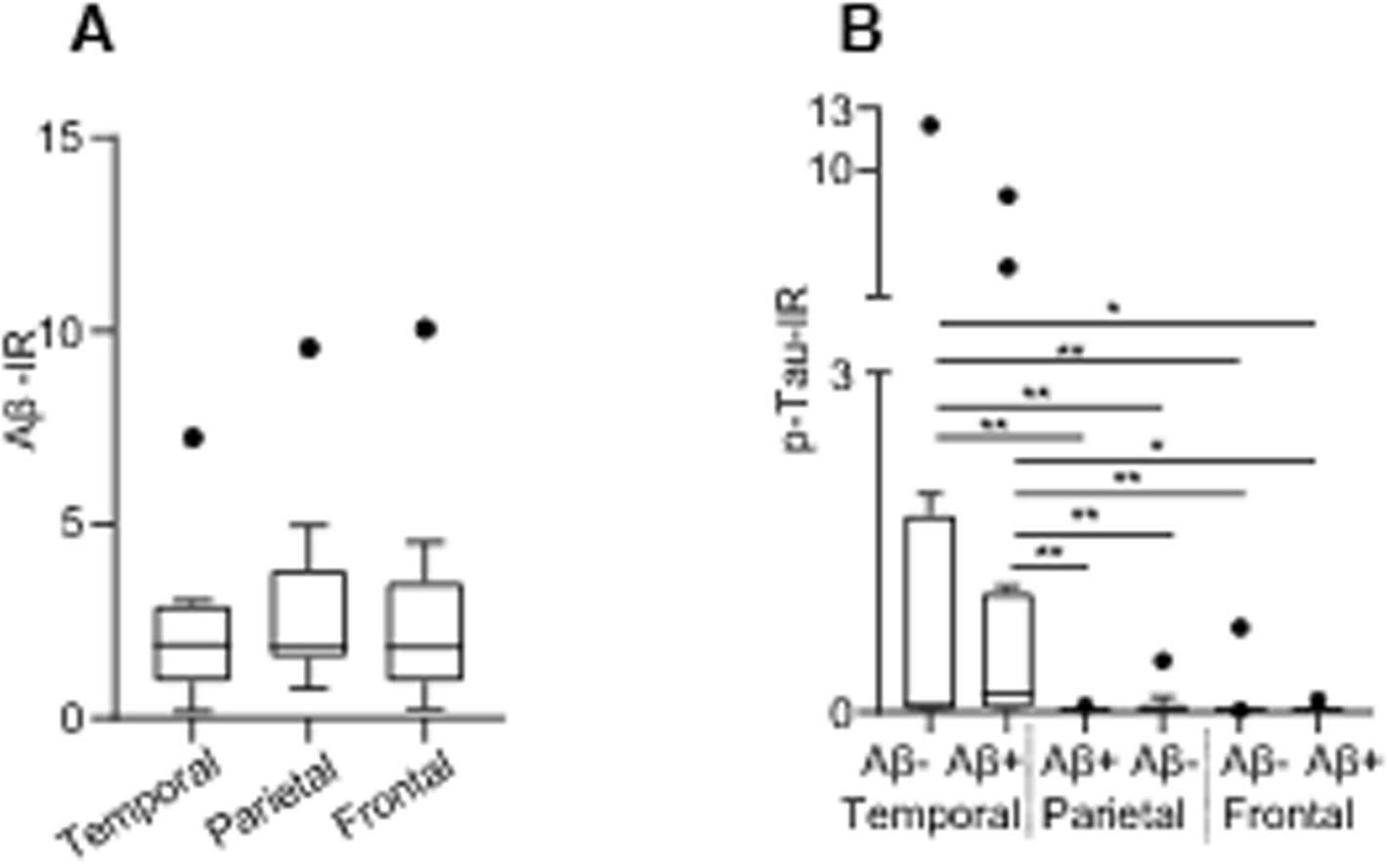
Aβ and tau deposition in the cortices of non-demented individuals with and without Aβ pathology. **a** Aβ deposition did not differ between the temporal, parietal, and frontal cortices of non-demented individuals harboring AD pathology; Kruskal–Wallis test, H = 0.496, *p = 0.78*. **b** The temporal cortex of Aβ− and Aβ+ individuals displayed increased tau pathology when compared with the parietal and frontal cortices; Kruskal–Wallis test, H = 14.94, *p < 0.0001* followed by Dunn’s multiple comparisons test. Data are displayed in boxplots where the median is represented by the horizontal line and the whiskers represent the 25th and 75th percentiles. Full circles represent outliers according to the Tukey method. *IR* immunoreactivity. ^*^p < 0.05, ^**^p < 0.01

Given the elevation of key inflammatory cytokines observed in the temporal, parietal, and frontal cortices analyzed, we examined whether there were differences in Aβ and p-tau deposition between these brain regions. No significant differences in Aβ deposition were observed between the temporal, frontal, and parietal cortices of Aβ+ individuals (*p = 0.780*) (Fig. 6a). In contrast, the temporal cortex of elderly individuals with or without Aβ deposition displayed higher levels of p-tau than the parietal and frontal cortices (*p < .0001*) (Fig. 6b).

## Discussion

**Fig. 7:**
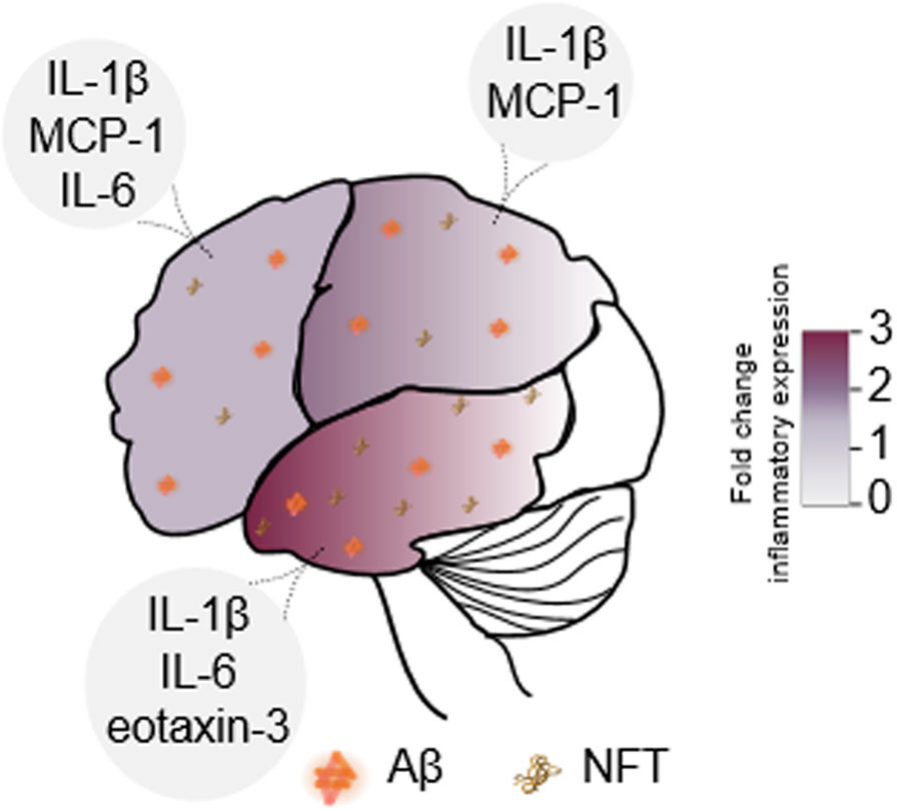
Brain cytokine expression, Aβ pathology, and tau deposition in non-demented individuals with cerebral Aβ pathology**.** The average fold change of inflammatory molecule expression per brain region was calculated. Briefly, NCI individuals with Aβ deposition displayed an increase in IL-1β (~ 1.8 fold change), IL-6 (~ 3.4 fold change), and eotaxin-3 (~ 3 fold change) in the temporal cortex; an increase in IL-1β (~ 2 fold change) and MCP-1 (~ 1.8 fold change) in the parietal cortex; and similar trends revealing higher levels of IL-1β (~ 1.5 fold change), IL-6 (~ 1.8 fold change), and MCP-1 (~ 1.7 fold change) in the frontal cortex as compared with individuals without Aβ deposition. Tau pathology was highest in the temporal cortex as compared with the parietal and frontal cortices from Aβ+ individuals. Individuals with Aβ pathology also showed cognitive deficits in perceptual speed

In this study, we show that non-demented individuals with cortical Aβ deposition display cognitive impairments in specific cognitive domains, such as perceptual speed, suggesting that such cognitive domain might be disrupted at early stages in the AD continuum. This is in line with previous studies reporting the occurrence of cognitive impairments in NCI individuals with Aβ pathology [17, 18]. Our studies also underscore that global cognitive scores might mask subtle cognitive domain-specific deficits. Furthermore, key inflammatory cytokines were differentially elevated across brain regions from elderly individuals with Aβ deposition (Fig. 7), reinforcing the notion of an early, preclinical neuroinflammatory process in the continuum of the AD pathology [1, 3, 4].

Notably, non-demented individuals with Aβ pathology had an overall increase in IL-1β in the three brain regions analyzed. Thus, raising the possibility that IL-1β might be one of the earliest inflammatory markers associated with the accumulation of Aβ neuropathology in older adults. IL-1β overexpression might contribute to potentiate AD neuropathology [19–21]. Early involvement of IL-1β in the AD continuum is also suggested by its presence in diffuse amyloid plaques in AD brains [22] and increased expression of caspase-1, which promotes IL-1β maturation, at prodromal and clinical stages of AD [23]. Moreover, IL-1β upregulation has been reported before the establishment of an overt AD pathology in Down syndrome [24, 25]. Increased neuronal IL-1β expression is present in a transgenic rat model of amyloidosis before amyloid plaque deposition [5] .

IL-1β expression may lead to the translation of IL-6 [26]. In our study, IL-6 levels were likewise increased in the temporal cortex of non-demented individuals with Aβ pathology. Similar to IL-1β, IL-6 is also present in diffuse amyloid plaques in AD brains [27] and can enhance AD pathology and cognitive deficits in animal models [28–30]. Importantly, genetic variations of the IL-6 and IL-1β genes have been associated with an increased risk of AD [31]. Of note, although IL-1β and IL-6 expression is often accompanied by increased TNF-α levels [32], we did not observe higher TNF-α levels in brains with Aβ deposition. In addition, the relatively low levels of TNF-α measured in this study, might mask subtle expression differences between brains with and without Aβ deposition. Therefore, more sensitive assays are needed to confirm whether TNF-α becomes dysregulated at early AD stages.

The chemokine eotaxin-3 was also elevated in the temporal cortex of AD-asymptomatic individuals with Aβ pathology. Increased eotaxin-3 levels have been reported in the CSF of individuals with AD dementia and can distinguish AD from other neurodegenerative diseases [33–35]. Moreover, eotaxin-3 levels are elevated before the establishment of overt AD pathology in the brains of people with Down syndrome [25]. It has also been reported that eotaxin-3 levels correlate with CSF tau pathology in AD [33]. Interestingly, in our study, eotaxin-3 was only upregulated in the temporal cortex of NCI individuals with Aβ pathology, and such region displayed higher levels of tau pathology than the parietal and frontal cortices. Therefore, higher eotaxin-3 levels might reflect an immune response to pathological tau in elders with amyloid deposition.

Increased levels of MCP-1 were found in the parietal cortex of individuals with Aβ. MCP-1 is a potent monocyte chemoattractant, and its upregulation in transgenic models of AD was suggested to contribute to peripheral monocyte infiltration into the brain [36, 37]. MCP-1 upregulation has been reported in AD brains and body fluids [38, 39]. Notably, MCP-1 neuronal levels are upregulated before the development of amyloid plaques in a transgenic rat model of the AD-like pathology and in Aβ-burdened neurons in the human cerebral cortex [9], supporting its early involvement at incipient stages of amyloid accumulation.

A trend towards higher levels of IL-1β, IL-6, and MCP-1 levels was observed in the frontal cortex of Aβ+ individuals. This suggests that altered inflammatory protein expression in the frontal cortex might occur after its exacerbation in the temporal and parietal cortices. It is also possible that the small sample size posed a limitation to detect significant differences in this cortical region. Therefore, these analyses remain exploratory and await to be confirmed in a larger and independent dataset.

Our study also showed that although amyloid levels did not significantly differ among brain regions, the temporal cortex displayed higher tau deposition and inflammatory cytokine expression than the parietal and frontal cortices. Therefore, it might be possible that the combined tau and amyloid pathologies make the temporal cortex a more vulnerable region to increased inflammatory protein expression.

In accordance with our results, cognitively normal individuals with CSF Aβ pathological values displayed an upregulation of CSF inflammatory and cerebrovascular markers compared with NCI individuals with normal CSF Aβ values [40]. In addition, astroglial and microglial activation are already present before the onset of dementia in populations at genetic risk of AD [25, 41]. Importantly, AD-resilient brains display decreased microglia and astroglia activation and anti-inflammatory and pro-resolution cytokine signature [42, 43]. Moreover, “SuperAgers” brains also display lower levels of microglial activation, comparable to those found in young individuals [44]. Besides the expression of inflammatory cytokines, other pathological events have been reported to occur in the brains of cognitively normal individuals harboring AD neuropathology such as synaptic deficits [45], oxidative stress [45], growth factor dysregulation [46], and epigenetic modifications [47]. Further experiments are needed to understand whether these and the previously mentioned inflammatory changes are also present in brains with or without Aβ pathology.

In this study, none of the upregulated cytokines in the temporal, parietal, or frontal cortices was associated with the rate of cognitive decline. It is important to note that longitudinal cognitive measures and cytokine brain expression analyses are disconnected in the time of assessment. Therefore, this type of analyses would have more informative value if cytokine expression was measured in cerebrospinal fluid followed by post-mortem assessment of cytokine brain expression at the time of death.

Surprisingly, our results also show that some cytokines that display similar expression levels in brains with and without Aβ deposition, correlate either positively or negatively with the rate of cognitive decline in specific cognitive domains. While higher cytokine expression (IL-1α, IL-13, and MDC) in the temporal cortex was associated with worsening of cognitive function, the opposite was true for cytokine levels in the parietal (MIP-1β and TARC) and frontal (IL-16 and IP-10) cortices. These observations might reflect the transient nature of cytokine expression and its relationship with AD progression [48]. Moreover, the negative associations between cytokine expression in the temporal cortex and cognitive deficits might be associated with the increased tau pathology that was observed in this region. However, these complex exploratory observations warrant further comprehensive longitudinal investigations to elucidate whether cytokine expression exerts transient effects on cognition at different stages of the AD continuum.

This study has several limitations that must be considered. First, although post-mortem pathological analysis of AD-asymptomatic individuals indicated the presence of pathology meeting AD criteria, we cannot ascertain whether these individuals would have progressed through the AD continuum had they lived longer. However, the faster cognitive decline in elderly individuals with Aβ deposition, the higher proportion of APOE e4 individuals in this population, and the neuropathological diagnosis of AD according to modified NIA-Reagan and CERAD criteria, would suggest that these individuals might have indeed been at the earliest stages of AD. Second, to study preclinical stages of AD and to avoid confounding factors (such as the presence of Super-Agers), we conducted our investigations in NCI individuals under age 80. Such criteria limited the number of samples from the ROS and ROSMAP cohorts available for analysis. Although the groups in our study were sex-balanced, the restricted sample size was not sufficient to investigate the effects of sex on cytokine expression and cognition. Moreover, the narrow sample analyzed in this study could have limited the detection of subtle cognitive deficiencies in other cognitive domains. Ideally, this study should be confirmed in a larger sample size, when available. Finally, we have segregated our population by the extent of extracellular Aβ deposition. Besides being a highly selected sample, we acknowledge that there is not a clear “cutoff” value between what represents healthy and AD-related Aβ deposition. Moreover, to fully understand the earliest inflammatory changes associated with preclinical AD, it would be important to investigate the neuroinflammatory response associated with early intraneuronal Aβ accumulation. Equally important would be to measure soluble Aβ levels in brains with and without Aβ deposition to define the Aβ concentration threshold associated with cytokine dysregulation.

## Conclusions

Our findings provide a link between amyloid deposition, key brain inflammatory cytokines, and cognitive decline in non-demented elderly individuals with amyloid accumulation. Our study also suggests that stratification by amyloid positivity should be considered when evaluating anti-inflammatory approaches for AD. The investigation of cognitively healthy individuals with AD neuropathology offers the possibility of identifying the earliest neuropathological changes associated with preclinical AD, allowing for the identification of novel therapeutic targets and biomarker candidates.

## Supplementary Information

**Additional file 1: Supplemental Table 1.** Demographics of the studied population: temporal cortical samples from elderly individuals.

**Additional file 2: Supplemental Table 2.** Demographics of the studied population: parietal cortical samples from elderly individuals.

**Additional file 3: Supplemental Table 3.** Demographics of the studied population: frontal cortical samples from elderly individuals.

**Additional file 4: Supplemental Figure 1.** Amyloid-beta and tau neuropathology in brains with Aβ deposition. Figure legend: Representative micrographs depicting amyloid-beta and tau pathology as revealed by immunohistochemistry (IHC) with 4G8 and AT8 antibodies, respectively. (A-B) Representative micrographs of cases with Aβ deposition in the temporal, parietal and frontal cortices. CV = Cresyl violet counterstaining. Scale bar = 100μm.

**Additional file 5: Supplemental Table 4.** Association of cognitive scores with global amyloid and tau levels.

**Additional file 6: Supplemental Table 5.** Correlation of post-mortem interval with cytokine expression in the temporal, parietal and frontal cortices.

**Additional file 7: Supplemental Table 6.** Association of cytokine expression with cortical amyloid-beta and pathological tau burden in the temporal, parietal and frontal cortices of non-demented elderly individuals.

**Additional file 8: Supplemental Table 7.** Association of cognitive decline with cytokine expression in the temporal, parietal and frontal cortices of non-demented elderly individuals.

## Data Availability

The datasets used and/or analyzed during the current study are available from the corresponding author on reasonable request.

## Abbreviations

AD: Alzheimer’s disease
Aβ: Amyloid-beta
CERAD: Consortium to Establish a Registry for Alzheimer’s Disease
IFN: Interferon
IL: Interleukin
IP-10: Interferon-inducible protein-10,
MAP: Rush Memory and Aging Project
MCP-1: Monocyte chemoattractant protein
MDC: Macrophage-derived chemokine
MIP-1β: Macrophage inflammatory protein
NCI: No cognitive impairment
NIA: National Institute on Aging
ROS: Religious Orders Study
TARC: Thymus and activation-regulated chemokine
TNF: Tumor necrosis factor
VEGF-A: Vascular endothelial growth factor A
