# Supplementary material for: Cognitive and brain cytokine profile of non-demented individuals with cerebral amyloid-beta deposition
Source: J Neuroinflammation. 2021 Jul 4;18:147. doi: 10.1186/s12974-021-02169-0 (PMC8254948; doi:10.1186/s12974-021-02169-0)
Supplement: Supplementary file 1 — Additional file 1: Supplemental Table 1. Demographics of the studied population: temporal cortical samples from elderly individuals. [file 12974_2021_2169_MOESM1_ESM.docx]

**Supplemental Table 1.** Demographics of the studied population: temporal cortical samples from elderly individuals

| **Group** |  | **Aβ -** | **Aβ +** | ***p-value*** |
| --- | --- | --- | --- | --- |
| **Sample size** |  | n=16 | n=12 |  |
| **Age** | range (years) | 66.21-79.53 | 69.65-79.63 |  |
|  | mean ± SD | 75.10 ± 4.017 | 76.19 ± 3.161 | *0.446* |
| **Sex** | (F/M) | 7/9 | 7/5 | *0.703* |
| **PMI** | range (hours) | 3-18.67 | 2.5-29.58 |  |
|  | mean ± SD | 10.18 ± 5.367 | 8.562 ± 8.829 | *0.080* |
| **Cogn global** | mean ± SD | 0.3365 ± .3974 | 0.3897 ± .3298 | *0.251* |
| **Years of education** | mean ± SD | 17.88 ± 3.897 | 18 ± 3.191 | *0.92* |
| **Apoe4** |  | 0 | 3 (25%) | *0.05* |
| **ApoE distribution** | ε 2/2  ε 2/3  ε 2/4  ε 3/3  ε 3/4  ε 4/4 | 0  4 (25%)  0  12 (75%)  0  0 | 0  0  0  9 (75%)  3 (25%)  0 |  |
| **Braak score** | 0 – II  III - IV | 9 (60 %)  6 (40 %) | 4 (33.33%)  8 (66.67%) | *0.25* |
| **Braak score distribution** | 0  I  II  III  IV  V  VI | 1 (6.25%)  5 (31.25%)  4 (25%)  5 (31.25%)  1 (6.25%)  0  0 | 0  3 (25%)  1 (8.33%)  4 (33.33%)  4 (33.33%)  0  0 |  |
| **CERAD** | possible or no AD  probable or definite AD | 16 (100 %)  0 | 3 (25%)  9 (75%) | ***<0.0001*** |
| **CERAD distribution** | no AD  possible AD  probable AD  definite AD | 15 (93.75%)  1 (6.25%)  0  0 | 3 (25%)  0  8 (66.67%)  1 (8.33%) |  |
| **NIA-Reagan** | low or no likelihood  intermediate/high likelihood | 16 (100%)  0 | 5 (41.66%)  7 (58.33%) | ***0.0007*** |
| **NIA-Reagan distribution** | no likelihood  low likelihood  intermediate likelihood  high likelihood | 1 (6.25%)  15 (93.75%)  0  0 | 0  5 (41.66%)  7 (58.33%)  0 |  |
| **p-tau** | mean ± SD | 1.244 ± 3.122 | 1.439 ± 2.778 | *0.463* |
| **Aβ-IR** | mean ± SD | 0 | 2.23 ± 1.847 |  |

Abbreviations: PMI = *post-mortem* interval, Cogn = cognition, Aβ = amyloid beta, F = female, M = male, IR = immunoreactivity, SD = standard deviation. Data are presented as mean ± SD
