# Supplementary material for: Cognitive and brain cytokine profile of non-demented individuals with cerebral amyloid-beta deposition
Source: J Neuroinflammation. 2021 Jul 4;18:147. doi: 10.1186/s12974-021-02169-0 (PMC8254948; doi:10.1186/s12974-021-02169-0)
Supplement: Supplementary file 2 — Additional file 2: Supplemental Table 2. Demographics of the studied population: parietal cortical samples from elderly individuals. [file 12974_2021_2169_MOESM2_ESM.docx]

**Supplemental Table 2.** Demographics of the studied population: parietal cortical samples from elderly individuals

| Group |  | Aβ - | Aβ + | *p-value* |
| --- | --- | --- | --- | --- |
| Sample size |  | n = 15 | n = 12 |  |
| Age | range (years) | 67.37-79.53 | 69.65-79.63 |  |
|  | mean ± SD | 75.99± 3.268 | 76.28 ± 3.21 | *0.718* |
| Sex | (F/M) | 6/9 | 7/5 | *0.449* |
| PMI | range | 2.5-18.67 | 2.5-29.58 |  |
|  | mean ± SD | 9.352 ± 5.536 | 9.167 ± 8.622 | *0.379* |
| Cogn global | mean ± SD | .389 ± .366 | .366 ± .3519 | *0.867* |
| Years of education | mean ± SD | 17.07 ± 3.918 | 18.67 ± 2.605 | *0.551* |
| Apoe4 |  | 1 (6.67%) | 3 (25 %) | *0.294* |
| ApoE distribution | ε 2/2  ε 2/3  ε 2/4  ε 3/3  ε 3/4  ε 4/4 | 0  3 (20 %)  0  11 (73.33 %)  1 (6.66 %)  0 | 0  0  0  9 (75%)  3 (25%)  0 |  |
| Braak score | 0 – II  III - IV | 10 (66.67 %)  5 (33.33 %) | 4 (33.33%)  8 (66.67%) | *0.128* |
| Braak score distribution | 0  I  II  III  IV  V  VI | 1 (6.66 %)  6 (40 %)  3 (20 %)  4 (26.66 %)  1 (6.66 %)  0  0 | 0  2 (16.66 %)  2 (16.66 %)  4 (33.33%)  4 (33.33%)  0  0 |  |
| CERAD | possible or no AD  probable or definite AD | 15 (100 %)  0 | 3 (25%)  9 (75%) | ***< 0.0001*** |
| CERAD distribution | no AD  possible AD  probable AD  definite AD | 14 (93.33 %)  1 (6.66 %)  0  0 | 2 (16.66 %)  1 (8.33 %)  8 (66.67%)  1 (8.33%) |  |
| NIA-Reagan | low or no likelihood  intermediate/high likelihood | 15 (100%)  0 | 5 (41.66%)  7 (58.33%) | ***0.0009*** |
| NIA-Reagan distribution | no likelihood  low likelihood  intermediate likelihood  high likelihood | 1 (6.66%)  14 (93.33%)  0  0 | 0  5 (41.66%)  7 (58.33%)  0 |  |
| p-tau | mean ± SD | .006 ± .014 | .057 ± .1338 | *0.853* |
| Aβ-IR | mean ± SD | 0 | 2.901 ± 2.434 |  |

Abbreviations: PMI = *post-mortem* interval, Cogn = cognition, Aβ = amyloid beta, F = female, M = male, IR = immunoreactivity, SD = standard deviation. Data are presented as mean ± SD
