# Supplementary material for: Cognitive and brain cytokine profile of non-demented individuals with cerebral amyloid-beta deposition
Source: J Neuroinflammation. 2021 Jul 4;18:147. doi: 10.1186/s12974-021-02169-0 (PMC8254948; doi:10.1186/s12974-021-02169-0)
Supplement: Supplementary file 3 — Additional file 3: Supplemental Table 3. Demographics of the studied population: frontal cortical samples from elderly individuals. [file 12974_2021_2169_MOESM3_ESM.docx]

**Supplemental Table 3.** Demographics of the studied population: frontal cortical samples from elderly individuals

| Group |  | Aβ - | Aβ + | *p-value* |
| --- | --- | --- | --- | --- |
| Sample size |  | n=13 | n=13 |  |
| Age | range (years) | 72.25-79.53 | 69.65-79.63 |  |
|  | mean ± SD | 76.97± 1.97 | 75.74 ± 3.378 | *0.271* |
| Sex | (F/M) | 5/8 | 5/8 | *>0.999* |
| PMI | range | 2.5-18.22 | 2.5-29.58 |  |
|  | mean ± SD | 8.971 ± 5.175 | 9.865 ± 8.283 | *0.715* |
| Cogn global | mean ± SD | .4083 ± .3721 | .3302 ± .3828 | *0.603* |
| Years of education | mean ± SD | 17 ± 3.582 | 18.54 ± 2.602 | *0.598* |
| Apoe4 |  | 1 (7.69%) | 3 (23.08 %) | *0.593* |
| ApoE distribution | ε 2/2  ε 2/3  ε 2/4  ε 3/3  ε 3/4  ε 4/4 | 0  2 (15.38 %)  0  10 (76.92 %)  1 (7.69 %)  0 | 0  1 (7.69 %)  0  9 (69.23 %)  3 (23.07 %)  0 |  |
| Braak score | 0 – II  III - IV | 9 (69.23 %)  4 (30.77 %) | 5 (38.46 %)  8 (61.54 %) | *0.237* |
| Braak score distribution | 0  I  II  III  IV  V  VI | 1 (7.69 %)  5 (38.46 %)  3 (23.07 %)  3 (23.07 %)  1 (7.69 %)  0  0 | 0  2 (15.38 %)  3 (23.07 %)  5 (38.46 %)  3 (23.07 %)  0  0 |  |
| CERAD | possible or no AD  probable or definite AD | 13 (100 %)  0 | 4 (30.77 %)  9 (69.23 %) | ***0.0005*** |
| CERAD  distribution | no AD  possible AD  probable AD  definite AD | 12 (92.31 %)  1 (7.69 %)  0  0 | 3 (23.1%)  1 (7.69 %)  8 (61.54 %)  1 (7.69 %) |  |
| NIA-Reagan | low or no likelihood  intermediate/high likelihood | 13 (100%)  0 | 7 (53.85%)  6 (46.15%) | ***0.0149*** |
| NIA-Reagan distribution | no likelihood  low likelihood  intermediate likelihood  high likelihood | 1 (7.69%)  12 (92.30%)  0  0 | 0  7 (53.84%)  6 (46.15%)  0 |  |
| p-tau | mean ± SD | .0602 ± .2073 | .01097 ± .02948 | *0.948* |
| Aβ-IR | mean ± SD | 0 | 2.668 ± 2.573 |  |

Abbreviations: PMI = *post-mortem* interval, Cogn = cognition, Aβ = amyloid beta, F = female, M = male, IR = immunoreactivity, SD = standard deviation. Data are presented as mean ± SD
