# Supplementary material for: Cognitive and brain cytokine profile of non-demented individuals with cerebral amyloid-beta deposition
Source: J Neuroinflammation. 2021 Jul 4;18:147. doi: 10.1186/s12974-021-02169-0 (PMC8254948; doi:10.1186/s12974-021-02169-0)
Supplement: Supplementary file 5 — Additional file 5: Supplemental Table 4. Association of cognitive scores with global amyloid and tau levels. [file 12974_2021_2169_MOESM5_ESM.docx]

**Supplemental Table 4.** **Association of** **cognitive scores with global amyloid and tau levels.**

| **Cognition** | **Aβ_global_-IR**  **(r; p-value)** | **Tau_global_-IR**  **(r; p-value)** |
| --- | --- | --- |
| **Global** | 0.049; *0.795* | 0.097; *0.607* |
| decline | -0.205; *0.293* | 0.006; *0.973* |
| **Perceptual speed** | -0.105; *0.577* | -0.001; *0.994* |
| decline | **-0.377; *0.047*** | -0.132; *0.501* |
| **Episodic memory** | -0.039; *0.834* | 0.308; *0.097* |
| decline | 0.050; *0.797* | 0.189; *0.335* |
| **Working memory** | -0.120; *0.527* | 0.023; *0.900* |
| decline | -0.103; *0.600* | -0.025; *0.899* |
| **Semantic memory** | -0.007; *0.967* | -0.199; *0.291* |
| decline | -0.078; *0.689* | 0.127; *0.517* |
| **Visuospatial ability** | 0.065; *0.729* | 0.270; *0.148* |
| decline | 0.251; *0.197* | 0.033; *0.866* |

According to data normality, Pearson’s or Spearman’s correlation coefficients were assessed in correlation analyses. The decline score represents the estimated slopes reflecting the person-specific rate of cognitive decline (mean time of follow-up was 5.7 ± 3.6 years, interquartile range: 3 – 9 years. IR = immunoreactivity. Aβ-, *n = 14*, Aβ+, *n = 16.*
